# Supplementary material for: Strengthening evidence for text-based telehealth in post-operative care: A pragmatic study of the reach and effectiveness of two-way, text-based follow-up after voluntary medical male circumcision in South Africa
Source: PLoS One. 2025 Oct 3;20(10):e0314436. doi: 10.1371/journal.pone.0314436 (PMC12494287; doi:10.1371/journal.pone.0314436)
Supplement: S1 File — (DOCX) [file pone.0314436.s001.docx]

**Appendix x: Standards for Reporting Implementation Studies: the StaRI Checklist of items reported**

| **Checklist item** | | **Implementation strategy** | **Intervention†** | **Comments** |
| --- | --- | --- | --- | --- |
| **Title** | **1** | Identification as an implementation study, and description of the methodology in the title and/or keywords | | The title identifies the study as “a pragmatic study of the reach and effectiveness of two-way text-based follow-up…” |
| **Abstract** | **2** | Identification as an implementation study, including a description of the implementation strategy to be tested, the evidence-based intervention being implemented, and defining the key implementation and health outcomes | | Page 2, Line #28 identifies this as an implementation study Page 2, Lines #28-29 identifies the implementation strategy to be tested  Page 2, Lines #31-36 identifies the evidence-based intervention being implemented,  Page 2, Lines #37-40 defines the key implementation and health outcomes |
| **Introduction** | **3** | Description of the problem, challenge, or deficiency in healthcare or public health that the intervention being implemented aims to address | | Page 4, Lines #60-68 describes the problem, challenge, or deficiency in healthcare or public health that the intervention being implemented aims to address |
|  | **4** | The scientific background and rationale for the implementation strategy (including any underpinning theory, framework, or model, how it is expected to achieve its effects, and any pilot work) | The scientific background and rationale for the intervention being implemented (including evidence about its effectiveness and how it is expected to achieve its effects) | Page 4, Lines #68-79 describes the scientific background and rationale for the implementation strategy  Page 5, Lines #81-93 describes the scientific background and rationale for the intervention being implemented |
| **Aims and objectives** | **5** | The aims of the study, differentiating between implementation objectives and any intervention objectives | | Page 5, Lines #95-100 outlines the aim and objectives of the study |
| **Methods: description** | **6** | The design and key features of the evaluation (cross referencing to any appropriate methodology reporting standards) and any changes to study protocol, with reasons | | Pages 7-8, Lines #138-153 describes the design and key features of the evaluation |
|  | **7** | The context in which the intervention was implemented (consider social, economic, policy, healthcare, organisational barriers and facilitators that might influence implementation elsewhere) | | Page 8, Lines #155-161 describes the context in which the intervention was implemented |
|  | **8** | The characteristics of the targeted “site(s)” (locations, personnel, resources, etc) for implementation and any eligibility criteria | The population targeted by the intervention and any eligibility criteria | Pages 6-7, Lines #120-135 describes the characteristics of the targeted “site(s)”  Pages 11-12, Lines #213-223 describes the population targeted by the intervention |
|  | **9** | A description of the implementation strategy | A description of the intervention | Pages 6-7, Lines #120-135 describes the implementation strategy  Pages 9-10, Lines #183-209 describes the intervention |
|  | **10** | Any subgroups recruited for additional research tasks, and/or nested studies are described | | There were no subgroups recruited for additional research tasks and no nested studies |
| **Methods: evaluation** | **11** | Defined pre-specified primary and other outcome(s) of the implementation strategy, and how they were assessed. Document any pre-determined targets | Defined pre-specified primary and other outcome(s) of the intervention (if assessed), and how they were assessed. Document any pre-determined targets | Page 13, Lines #237-248 defines pre-specified primary and other outcome(s) of the implementation strategy, and how they were assessed.  Page 13, Lines #248-250 defines pre-specified primary and other outcome of the intervention, and how it was assessed. |
|  | **12** | Process evaluation objectives and outcomes related to the mechanism(s) through which the strategy is expected to work | | Not applicable |
|  | **13** | Methods for resource use, costs, economic outcomes, and analysis for the implementation strategy | Methods for resource use, costs, economic outcomes, and analysis for the intervention | Costs, economic outcomes, and analysis for the implementation strategy have been published elsewhere.  Costs, economic outcomes, and analysis for the intervention are addressed in another publication |
|  | **14** | Rationale for sample sizes (including sample size calculations, budgetary constraints, practical considerations, data saturation, as appropriate) | | Pages 12-13, Lines #225-235 describes the power and sample size. Table 2 on page 13 summarises the power and sample size calculations |
|  | **15** | Methods of analysis (with reasons for that choice) | | Pages 14-15, Lines #260-273 describes methods of analysis |
|  | **16** | Any a priori subgroup analyses (such as between different sites in a multicentre study, different clinical or demographic populations) and subgroups recruited to specific nested research tasks | | Not applicable. This was not a multicentre study |
| **Results** | **17** | Proportion recruited and characteristics of the recipient population for the implementation strategy | Proportion recruited and characteristics (if appropriate) of the recipient population for the intervention | Pages 17-18, Lines #307-315 Proportion recruited and characteristics of the recipient population for the implementation strategy  Table 4 on Pages 17-18, Lines #316-317 Proportion recruited and characteristics of the recipient population for the intervention |
|  | **18** | Primary and other outcome(s) of the implementation strategy | Primary and other outcomes of the intervention (if assessed) | Pages 16-20, Lines #300-347 describes primary outcomes of the intervention.  Pages 20-24, Lines #349-395 describes secondary outcomes of the intervention |
|  | **19** | Process data related to the implementation strategy mapped to the mechanism by which the strategy is expected to work | | We connected data on the outcomes (AEs) to those who responded via 2wT, for instance, we included a table with all the AEs and whether they were ascertained through 2wT or SOC. |
|  | **20** | Resource use, costs, economic outcomes, and analysis for the implementation strategy | Resource use, costs, economic outcomes, and analysis for the intervention | This was assessed in a separate costing study |
|  | **21** | Representativeness and outcomes of subgroups including those recruited to specific research tasks | | N/A. We did not assess subgroups. However, we did assess characteristics of those who chose WhatsApp vs SMS in a separate paper. https://pubmed.ncbi.nlm.nih.gov/39412842/ |
|  | **22** | Fidelity to implementation strategy as planned and adaptation to suit context and preferences | Fidelity to delivering the core components of intervention (where measured) | Fidelity to delivering the core components of intervention was not measured |
|  | **23** | Contextual changes (if any) which may have affected outcomes | | There were no contextual changes that affected outcomes |
|  | **24** | All important harms or unintended effects in each group | | N/A |
| **Discussion** | **25** | Summary of findings, strengths and limitations, comparisons with other studies, conclusions and implications | | Page 24, Lines #398-411 provides summary of findings, strengths and limitations, comparisons with other studies, conclusions and implications |
|  | **26** | Discussion of policy, practice and/or research implications of the implementation strategy (specifically including scalability) | Discussion of policy, practice and/or research implications of the intervention (specifically including sustainability) | Pages 24-25, Lines #413-428 discusses policy, practice and/or research implications of the implementation strategy  Pages 25-28, Lines #430-488 discusses policy, practice and/or research implications of the intervention |
| **General** | **27** | Include statement(s) on regulatory approvals (including, as appropriate, ethical approval, confidential use of routine data, governance approval), trial or study registration (availability of protocol), funding, and conflicts of interest | | Page 15, Lines #275-282 include statement on regulatory approvals, trial or study registration  Pages 30-31, Lines #534-547 include statement on funding,  Pages 31, Lines #549-550 include statement on conflicts of interest |

*Implementation strategy refers to how the intervention was implemented.

†Intervention refers to the healthcare or public health intervention that is being implemented.

Note: A key concept is the dual strands of describing (*a*) the implementation strategy and (*b*) the clinical, healthcare, or public health intervention that is being implemented. These strands are represented as two columns in the checklist. The primary focus of implementation science is the implementation strategy (column 1) and the expectation is that this will always be completed. The evidence about the impact of the intervention on the targeted population should always be considered (column 2) and either health outcomes reported or robust evidence cited to support a known beneficial effect of the intervention on the health of individuals or populations. While all items are worthy of consideration, not all items will be applicable to or feasible within every study.
